# Impact of Drying-Induced Structural Modifications on Flavor Release of Star Anise During Boiling

**DOI:** 10.3390/foods14101802

**Published:** 2025-05-19

**Authors:** Xiangmin Kuang, Silei Zhang, Chaofan Guo, Yongli Jiang, Wenchao Liu, Fujie Zhang, Qingbo Huang, Junjie Yi

**Affiliations:** 1 Faculty of Food Science and Engineering, Kunming University of Science and Technology, Kunming 650500, China; 18287426229@163.com (X.K.); zhangsilei1228@163.com (S.Z.); guochaofanfan@outlook.com (C.G.); yongli_jiang0617@163.com (Y.J.); 2 Key Laboratory of Prefabrication of Plateau Specialty Food in Yunnan Province, Kunming 650500, China; 3 International Green Food Processing Research and Development Center of Kunming City, Kunming 650500, China; 4 College of Food and Biotechnology, Henan University of Science and Technology, Luoyang 471000, China; wen_chaoliu@163.com; 5 Faculty of Modern Agriculture Engineering, Kunming University of Science and Technology, Kunming 650500, China; 20030031@kust.edu.cn; 6 Funing Star Anise Research Institute, Funing 663400, China; 18314422948@163.com

**Keywords:** star anise, drying technique, flavor release, trans anethole, water migration

## Abstract

Star anise, a traditional seasoning, plays a significant role in influencing consumer preferences through its flavor release during cooking. This study examines how drying techniques—hot air drying (HAD), heat pump drying (HPD), Far-infrared drying (FIRD), and microwave vacuum drying (MVD)—affect the visual appearance and flavor release of star anise. Among these techniques, HAD required the longest drying time (20.5 h) and had the highest fracture rate (11.97%), while MVD achieved the shortest drying time (70 min) and FIRD had the lowest fracture rate (9.84%). Color analysis of dried star anise fruits revealed no significant differences among HAD, HPD, and FIRD (p* > 0.05), but MVD resulted in poorer color quality. Following boiling, 26 aroma compounds were identified, with trans-anethole and anisic aldehyde being the most prominent. Compared to other techniques, HPD exhibited the highest volatile oil content and trans-anethole levels in star anise after cooking. Overall, HPD produces star anise with superior visual quality and enhanced flavor release during cooking, making it a more suitable option for large-scale drying.

## 1. Introduction

Star anise (*Illicium verum Hook. f.*), a prominent spice and medicinal herb native to East Asia [1], particularly China and Vietnam, has garnered significant attention in both culinary and pharmaceutical industries worldwide [2]. This star-shaped fruit, known for its distinctive licorice-like flavor, belongs to the family *Schisandraceae*. It is commonly added to stews and hotpots to elevate the aroma and richness of the broth, thereby enhancing the overall flavor profile. Furthermore, adding star anise to stir-fried dishes helps mask any fishy odors from meat and seafood, balances greasy flavors, and stimulates gastric juice secretion, aiding digestion. However, the effects of flavor release from star anise during cooking have been insufficiently studied.

Drying is a crucial processing step for star anise, as drying technologies affects its flavor release [3]. Commercially, star anise is predominantly traded in dried form due to its extended shelf life. Conventional drying methods such as sun drying and hot air drying (HAD) remain widely used, but these techniques often lead to uneven drying, color degradation, and loss of volatile compounds. The dried product’s quality, including flavor retention and structural integrity, is critical for consumer acceptance and industrial applications. It is well-known that excessive temperatures during the drying process can degrade the aromatic components of star anise, leading to irreversible losses that negatively impact its flavor. The primary technologies currently used for drying star anise include heat air drying (HAD), heat pump drying (HPD), far-infrared drying (FIRD), and microwave vacuum drying (MVD), each with unique advantages. Regarding the four drying technologies, the HAD process employs convective heating to effectively evaporate moisture from materials, making it a reliable choice for bulk drying [4]; HPD enhances energy efficiency by utilizing ambient heat through advanced heat pump technology to transfer thermal energy into the drying chamber [5]; the FIRD process stands out by using far-infrared radiation for direct heating, which not only boosts drying efficiency but also preserves the quality of heat-sensitive materials; MVD integrates microwave energy within a vacuum environment, facilitating rapid and efficient moisture removal, thus improving product characteristics. Star anise is widely used in the cooking of various ingredients, such as soups, hot pots, stews, and braised meats. Studying the flavor-release capacity of star anise after cooking under different drying technologies can provide valuable references for its application in different culinary scenarios. This is particularly important because the flavor-extraction efficiency and release kinetics of star anise vary depending on cooking methods—slow simmering, rapid boiling, or prolonged stewing—all of which influence its final sensory contribution. Therefore, understanding how different drying methods affect its post-cooking flavor performance can help optimize its usage in specific dishes, ensuring consistent and desirable taste profiles. While some studies have examined the effects of different drying technologies on quality changes in star anise [6], the impact of drying on flavor release, especially during post-boiling cooking processes, has been underexplored.

To address this gap, this study aims to investigate the effects of various drying technologies, i.e., HAD, HPD, FIRD, and MVD, on the flavor release of star anise during boiling after drying. Initially, the drying rates and moisture migration characteristics associated with drying technologies were systematically analyzed. Subsequently, an examination of visual attributes, including color and fracture rates, alongside the volatile oil content of star anise dried, was conducted. Following this, the flavor release profiles of star anise post-boiling were assessed utilizing electronic nose (E-nose), electronic tongue (E-tongue), and gas chromatography-mass spectrometry (GC-MS) techniques. This research aimed to elucidate the mechanisms by which distinct drying methodologies impact the flavor-release potential of star anise, thereby offering valuable insights for future processing strategies and consumer selection of dried star anise.

## 2. Materials and Methods

### 2.1. Materials

Fresh star anise (*Illicium verum Hook. f.*) fruits were sourced from a local market in Funing, Yunnan Province, China. Only complete, uniformly sized specimens without mechanical damage were selected as the raw material for the study. Fresh star anise fruits were collected in two independent batches from the local market, which was divided into four experimental groups (HAD, HPD, FIRD, MVD), and three replicates per drying technique were performed.

### 2.2. Drying and Boiling Processing

The average initial moisture content of star anise, determined using the constant weight method, was found to be 78% (w. b). The following four drying techniques were applied to dry star anise until the moisture content reached around 15% (w. b). After drying, 5 g of star anise was weighed and added to 1L of water for boiling. This proportion is based on common cooking practices, which can ensure the flavor intensity and avoid excessive flavor. The detection of taste-related compounds release analysis was conducted by E-tongue during the boiling process (Section 2.8). Once boiling was complete, aroma profiles were analyzed using an E-nose and GC-MS (Section 2.9).

#### 2.2.1. Hot Air Drying

The HAD process was conducted following the method of [7]. Fresh star anise (300.0 ± 0.1 g) was taken and spread on a wire mesh tray. The process of HAD was conducted in a drying oven (101-3ES, Beijing, Yongguang Medical Device Factory, Beijing, China) at 60 °C with an air velocity of 1 m/s. The entire HAD process lasted for approximately 20.5 h, resulting in the sample reaching moisture equilibrium.

#### 2.2.2. Hot Pump Drying

The HPD drying process was conducted following the method of (Hou et al.) [8]. Fresh star anise, weighing 300.0 ± 0.1 g, was spread on a drying tray. This process was carried out in a drying oven (GHRH-20, Guangdong Agricultural Machinery Research Institute, Guangzhou, China) set at a temperature of 60 °C and 10% humidity. The entire HPD process lasted approximately 14 h, allowing the sample to reach moisture equilibrium.

#### 2.2.3. Far-Infrared Drying

Fresh star anise was spread onto steel girds uniformly in a far-infrared drying oven (YHW-20BE, Shanghai Juchen Scientific Instruments, Shanghai, China) with four infrared heating plates (266 W), set at 60 °C with a plate distance of 10 cm for 9 h (Shen et al.) [9].

#### 2.2.4. Microwave Vacuum Drying

The MVD process was conducted following the method of (Zhao et al.) [10]. Fresh star anise, weighing 300 ± 0.1 g, was placed in the vacuum chamber (JBL/BMW-2, Nanjing, Jingbaili Microwave, Nanjing, China). The sample was subjected to microwave treatment at a power density of 5 W/g, with the vacuum level reaching a maximum of −80 kPa. The desiccation process lasted approximately 70 min. Each drying process (HAD, HPD, FIRD, MVD) was executed in triplicate to ensure reproducibility.

### 2.3. Moisture Ratio Determination

During the drying process, the calculation of the moisture ratio was performed following the procedure reported by (Lu et al.) [11]. The dry basis moisture content of anise was calculated before conducting drying experiments, and it was calculated as Equation (1):

(1) $$X_{t}(g/g)=\frac{m_{t}-m}{m}$$

where *X*_t_ is the dry moisture content of the sample at time *t*; m_t_ is the mass of the sample at time t (g); m is the mass of the dry material (g).

Moisture ratio (moisture ratio, MR) was calculated as Equation (2):

(2) $$MR(kg/kg)=\frac{X_{t}-X_{e}}{X_{o}-X_{e}}$$

where *MR* is the moisture ratio; *X*_o_ is the dry basis moisture content of the sample at the initial moment; *Xe* is the dry basis moisture content of the sample at the equilibrium moment, representing the minimum moisture content the sample can achieve by the end of the drying process [12].

### 2.4. Low-Field Nuclear Magnetic Resonance

The detection method has been updated in accordance with the findings of (Bai et al.) [13]. Water status of dry star anise was conducted using a LF-NMR instrument (MesoMR23-060H-I, Niumag Corp., Shanghai, China). After weighing, the sample was placed in a 60-mm glass tube and inserted into the NMR probe. Carr–Purcell–Meiboom–Gill (CPMG) sequences were employed to measure spin–spin relaxation time, T2. The typical pulse parameters were as follows: Dwell time, 4 μs; echo time, 500.00 μs; recycle time, 600 ms; echo count, 1024; and scan repetitions, 16. The acquired transverse relaxation time T2 frequency images were inverted using software to generate the inversion spectra.

### 2.5. Color Measurement

Color determination was performed on dried star anise using a colorimeter (Agera, HunterLab) in reflectance mode. L* represents lightness, a* represents greenness to redness, and b* represents blueness to yellowness. ΔE* represents the total color difference, which is calculated using the following Equation (3). And the standard whiteboard (L_o_* = 93.2, a_o_* = −0.8, b_o_* = 0.4) as the reference.

(3) $$∆E=\sqrt{\left({∆L}^{*2}\right)+\left({∆a}^{*2}\right)+({∆b}^{*2})}$$

### 2.6. Fracture Rate and Rehydration Ratio

The fracture rate and rehydration ratio were calculated using the method reported by (Hu et al.) [14]. A random sample of 100 g of dried star anise was selected, and the broken lobes consisting of one to four segments were picked out with tweezers and weighed. The fracture rate was calculated using Equation (4).

(4) $$S=m_{2}/m_{1}\times 100\%$$

where *S* is the fracture rate; *m*_1_ is the total mass of the sample; *m*_2_ is the mass of the fractured segments.

The rehydration capacity of dried star anise was expressed as rehydration ratio. Dried anise was soaked in 200 mL of water at 60 °C for 30 min, then removed and drained for 5 min before weighing. The rehydration ratio was calculated using Equation (5):

*R* = *W_R_*/*W_D_* (5)

where *R* is the rehydration ratio of dried anise, *W_R_* is the mass of the sample after rehydration; *W_D_* is the mass of the sample before rehydration.

### 2.7. Scanning Electron Microscope Analysis

Scanning electron microscope (SEM) analysis was conducted on the star anise samples (Apreo™ 2 SEM, Thermo Fisher Scientiffc, Waltham, MA, USA). Cross-sections of the dried star anise samples were mounted on short stubs and sputter-coated with gold for 100 s. The microscope operated at 5 kV with a magnification of 800×.

### 2.8. Volatile Oil Content Determination

The volatile oil content was determined according to a previously reported method (Chai et al.) [15]. A 10 g sample of powdered star anise was placed in a 500 mL round-bottom flask, to which 200 mL of distilled water was added. The steam distillation apparatus was assembled, and the mixture was heated under reflux for a specified duration. The resulting distillate, consisting of a mixture of oil and water, was collected and subjected to salt precipitation before being transferred to a separator funnel. The volatile oil was then extracted twice using an organic solvent, with 15 mL of solvent each time. The organic layers were combined and dried over anhydrous sodium sulfate for 1 h. After drying, the solution was filtered, and the solvent was removed by evaporation. The volatile oil phase from the star anise was collected, weighed, and the extraction yield was calculated.

### 2.9. Aroma Profile Determination

#### 2.9.1. E-Nose Analysis

Odor analysis of boiled anise solution was performed following the methods of (Xie et al.) [16] with some modifications using a portable universal cNose (Baosheng Industrial Development Co., Ltd., Shanghai, China). The system consists of a sample device, a detector unit equipped with an array of 18 distinct metal oxide sensors, and pattern recognition software for data collection and analysis. 10 mL of the star anise infusion was transferred to a headspace vial, which was then sealed and allowed to stand for approximately 20 min to volatilize flavor compounds. The signal at 60 s was recorded for analysis, and the steady-state signal response value was used for interpretation. The samples were analyzed by headspace sampling at room temperature (24 ± 3 °C), with clean and dry air as the carrier gas at a flow rate of 1 L/min. The sampling interval was 1 s, with a sample connection time of 5 s, a cleaning time of 240 s, and a data acquisition time of 60 s [5]. The assay was carried out in triplicate.

#### 2.9.2. HS-SPME-GC-MS Analysis

The volatile compounds in each star anise water sample were analyzed using gas chromatography/mass spectrometry (GC/MS) in combination with headspace-solid phase microextraction (HS-SPME) as described by (Ye et al.) [17]. The volatile compounds were extracted using HS-SPME and analyzed using GC-MS at the Kunming University of Science and Technology Analysis and Testing Center (Yunnan province, China). Utilizing the SPME method, 5 mL of star anise water and 200 μL 3-octanol standard solution was precisely extracted into a 25 mL headspace vial and sealed. The extraction temperature was maintained at 50 °C. A pre-aged solid-phase microextraction fiber was inserted, positioned above the sample powder. After 40 min of extraction, the fiber was removed and promptly inserted into the injector port of the GC-MS system. Resorption was conducted at 250 °C for 10 s before GC-MS analysis. Chromatographic conditions involved a temperature program starting at 50 °C, held for 3 min, followed by an increase at a rate of 4 °C/min to 220 °C, with a final hold time of 10 min. The injector port and interface temperatures were maintained at 250 °C. High-purity helium (99.999%) served as the carrier gas at a flow rate of 1.0 mL/min. Mass spectrometric conditions comprised electron ionization (EI) at 70 eV, with interface and quadrupole temperatures set at 250 °C and 150 °C, respectively. The electron multiplier voltage was set at 2.28 kV, and the scan range spanned from m/z 35 to 500 emu.

Volatiles were identified by matching the experimental mass spectra with the standard spectra stored in NIST14 library data (a threshold match of 80%). Alkane mixture (C5-C25) was directly injected into GC-MS under the same operating conditions to calculate retention index (RI). Internal standard (3-Octanol) calibration was also conducted for semi-quantification. Analyses were performed using an Agilent 7890B GC coupled with a 5977B MSD (Agilent Technologies, California, Santa Clara, USA). Separation was achieved on an HP-INNOWAX capillary column (60 m × 0.25 mm × 0.25 μm). The model of HS-SPME is 65 μm pdms/dvb (Supelco, Bellefonte, PA, USA ).

### 2.10. Taste Profile Determination

Taste profile determination was carried out according to (Yin et al.) [18] with slight modifications using a C-Tongue system (Baosheng Industrial Development Co., Ltd., Shanghai, China). A schematic diagram of the device used to detect the release of taste-related compounds is shown in Figure S1. 10 mL of star anise water was extracted for electronic tongue (E-tongue) analysis. Subsequent analyses were conducted at 10-min intervals until the process was halted at 70 min. During electronic tongue detection, the probe sensitivity was diluted by a factor of 100.

### 2.11. Statistical Analysis

The mean and standard deviation of each set of data were calculated using SPSS Statistics 19 Version 13.0 (IBM, Chicago, IL, USA). One-way analysis of variance (ANOVA) was used, and significance was determined at *p* < 0.05 for the Tukey’s significant difference test that followed. Using Origin Pro software (version 8, Origin Lab Corporation, Northampton, MA, USA), hierarchical cluster analysis (HCA), and correlation analysis were carried out.

## 3. Results and Discussion

### 3.1. Moisture Ratio and Rehydration Ratio

#### 3.1.1. Moisture Ratio

**Figure 1:**
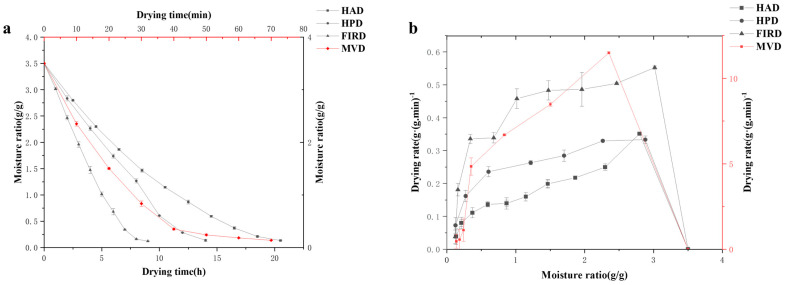
(**a**) The drying curve of star anise. (**b**) The drying rate curve of star anise.

The variation of drying rate with the change of dry basis moisture content is shown in Figure 1a,b. The curve indicates that the higher the moisture content of star anise, the higher the drying rate [19]. This is consistent with the overall drying process of star anise, which presents a decreasing drying rate over time. In the initial stage of drying, the moisture content of star anise was high, and the surface moisture rapidly evaporated, leading to a high drying rate. As the drying progressed, the surface moisture of star anise evaporated to a certain extent, and the internal moisture diffusion became the main part. Since the internal moisture was at a certain distance from the surface, there was resistance during diffusion, resulting in a gradual decrease in the drying rate.

When comparing the four drying technologies, the drying times are as follows: MVD (70 min) < FIRD (9 h) < HPD (14 h) < HAD (20.5 h). Compared with other HAD, HPD, and FIRD techniques, MVD has reduced the drying time by 17.67, 12.03, and 7.76 times, respectively, which has a significant advantage in the scene of rapid drying of star anise. The MVD utilizes microwave radiation to heat the substance internally while lowering the pressure in a vacuum, which accelerates moisture evaporation from star anise, resulting in significantly shorter drying times [20]. The FIRD employs infrared radiation to heat the surface of the material, rapidly evaporating moisture through conduction and convection to the interior, thus achieving faster drying compared to HAD and HPD [21]. In contrast, HAD operates by heating air and passing it through the star anise to remove moisture, primarily relying on convective heat transfer. Research by (Zhou et al.) [22] indicates that HAD requires longer durations to gradually eliminate moisture, resulting in relatively slower drying speeds. Overall, MVD and FIRD were portrayed as advanced techniques that enhance drying efficiency, making them superior choices for applications requiring rapid drying, while HAD was presented as a traditional method that may be suitable when longer drying times were acceptable.

#### 3.1.2. Rehydration Ratio

**Figure 2:**
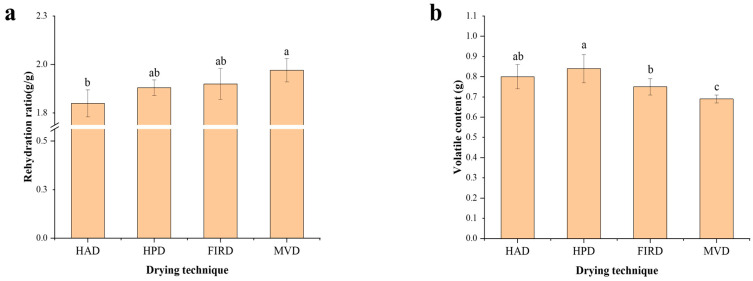
(**a**) The rehydration ratio of star anise. (**b**) The content of volatile oil. Different letters over bars (a, b, c) indicate that values belonging to those bars are significantly different (*p* < 0.05).

The rehydration capacity serves as an indicator of star anise’s ability to preserve internal structure integrity during drying. A higher rehydration capacity correlates with more intact internal structures and less drying-induced damage [23]. The rehydration ratio of star anise treated with the four drying techniques ranged from 1.8 to 1.97 (g/g), as illustrated in Figure 2a. The sample with MVD treatment had the strongest rehydration ability, while the sample with HAD treatment had the weakest rehydration ability. There is no significant difference in the rehydration ratio between FIRD and HPD samples (*p* > 0.05). This indicator was more favorable for anise treated under the MVD drying technique. (Ozcan-Sinir et al.) investigated various drying technologies for kumquats and found that samples dried using microwave drying exhibited higher rehydration capacity [24]. This suggests that microwave energy during drying might affect cellular interstitial spaces, enhancing water-absorption capability, thereby increasing the rehydration potential of star anise. The reason for the low rehydration rate of samples dried by hot air might be that HAD causes changes in the structure of star anise cell walls, which may become denser and reduce the permeability of water [25]. This made it difficult for water to effectively enter the cell interior during rehydration.

### 3.2. Visual Appearance of Star Anises Dried by Different Technologies

#### 3.2.1. Color

The color of dried star anise is a key indicator of its grade, with consumers favoring those that exhibit a dark brownish-reddish hue. Table 1 presents the L*, a*, b*, and ∆*E* for the four drying technologies applied to star anise. In comparison with the study of (Xu et al.) [26], dried star anise was similar in color under conventional thermal drying techniques (HAD, HPD). In this study, FIRD and MVD were added for comparison, and it was found that FIRD did not differ significantly from HAD and HPD (*p* > 0.05), and MVD differed significantly from all the first three (*p* < 0.05). Since MVD star anise has a higher b* and a lower a*, the color of the sample appears as yellowish green. The Δ*E* values for various dried star anise samples were calculated using the L*, a*, and b* values of the standard whiteboard as a reference. The range of Δ*E* from 64.82 to 68.64. There was a significant difference in MVD compared to the other three drying techniques (*p* < 0.05), but there was no significant difference between HAD, HPD, and FIRD (*p* > 0.05). This indicates that the visual differences between MVD samples and other samples could be clearly distinguished. This was likely due to the rapid drying process, which could prematurely deactivate enzymes within the star anise, preventing the development of enzymatic browning [8]. In contrast, HPD presents a better sensory state with its lower L* value and higher a* value.

#### 3.2.2. Fracture Rates

Star anise with intact eight corners and a lower fracture rate is generally more appealing to consumers, often correlating with a more intense flavor. As shown in Table 1, the highest is the HAD samples followed by HPD (10.79%) and MVD, while the fracture rate after FIRD was the lowest. During the drying process, the reason for the higher fracture rate of samples dried by HAD might be due to the presence of aerodynamic forces on the surface of the star anise during the drying process, which might cause the star anise to break and be damaged [27]. The fracture rate between MVD samples and HPD samples were moderate, and there was no significant difference between them (*p* > 0.05). The HPD systems typically involve thermal and moisture exchanges between the upper and lower sections, resulting in considerable fluctuations in surface temperature and humidity. This fluctuation may increase the risk of fractures [28]. The reason for the higher breakage rate under dry MVD conditions may be because it applies substantial pressure during the drying process, which may also contribute to damage [29]. In contrast, FIRD utilizes single-point infrared irradiation, exerting minimal impact on the star anise’s surface, and consequently results in the lowest fracture rate among the drying technologies [30].

### 3.3. Volatile Oil Content of Star Anises Dried by Different Technologies

Star anise has gained popularity as a widely used spice in cooking and food processing, owing to its abundant volatile oil content [31]. The primary constituents of star anise volatile oil included camphor, linalool, and eucalyptol, which contribute to enhancing the aroma of food [32].

Figure 2b illustrates the volatile oil content of star anise dried using four different technologies. The highest volatile oil content (0.84 mg/mL) was observed in HPD-treated star anise, aligning with the optimal volatile compound preservation reported by (Shi et al. 2021) for this drying technique, followed by HAD (0.79 mg/mL) and FIRD (0.75 mg/mL), while the content of volatile oil after MVD was the lowest (0.69 mg/mL) [6]. The reason for the highest concentration of volatile oil in star anise under HPD may be the enhancement is likely due to HPD operating at 60 °C, which enables efficient dehydration at relatively low temperatures, thereby preserving the volatile oils in the star anise. Conversely, MVD samples showed the lowest yield of volatile oil, potentially due to microwave-induced damage to certain internal structures of the star anise, leading to a decrease in oil content [33]. Additionally, in a vacuum setting, microwave heating can cause partial evaporation of volatile oils, although at a lower rate because of the reduced gas-phase pressure [34].

### 3.4. Flavor Release of Dried Star Anise During Boiling

As a commonly used spice in cooking, star anise is often added to stewed dishes to provide unique aroma and tastable compounds. Therefore, studying the tastable and aroma release patterns of star anise during boiling is of great significance for its application as a spice in practical processing.

#### 3.4.1. Aroma Release

**Figure 3:**
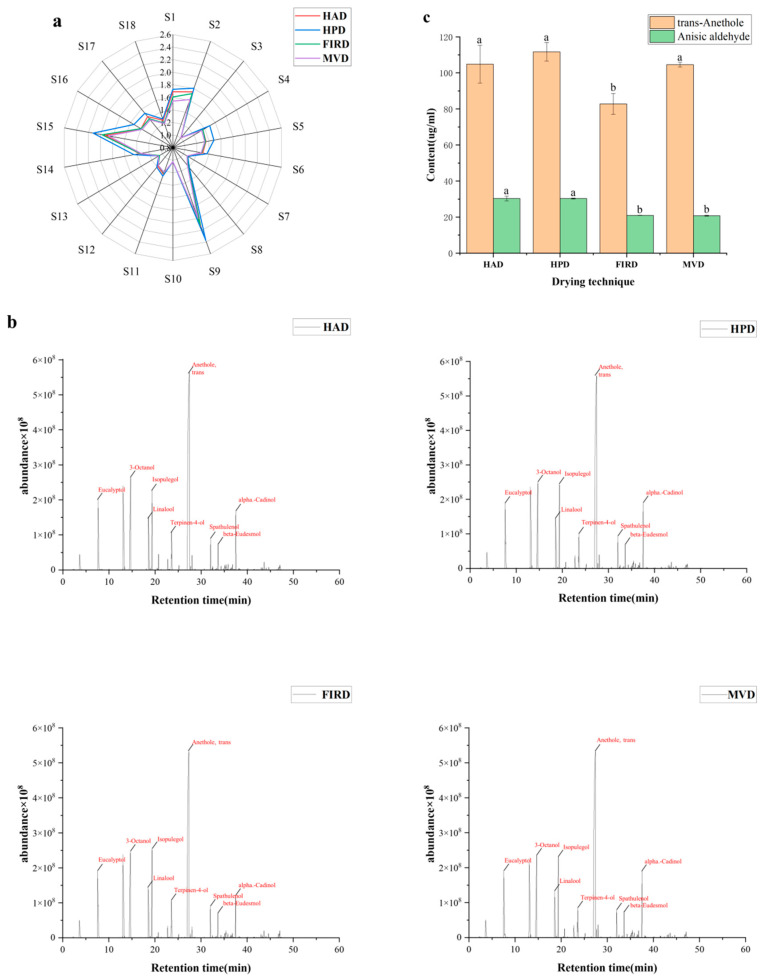
(**a**) Radar plot of electronic nose response values. (**b**) Representative total ion chromatogram of HS-SPME-GC–MS for star anise water. (**c**) The content of Trans anethole and Anisaldehyde. a, b: Different letters on different columns represent significant differences. (*p* < 0.05).

To better analyze the aroma compounds in star anise water, the overall distribution of aroma components was first explored using E-nose, and then the key aroma compounds were analyzed using GC-MS. As shown in the sensor configuration table of the E-nose in Figure 3a, the ion chromatogram analyzed by GC-MS of star anise water in Figure 3b Based on the radar response values from the electronic tongue.

Figure 3a reveals that the responses of sensors S9 and S15 were different among samples dried by different processing technologies, indicating the volatile compounds in star anise contain amounts of alkanes, alcohols, ketones, and other substances [35]. Furthermore, a comparison of different drying technologies demonstrates that the response values for star anise in the E-nose sensors vary depending on the drying technique used. Notably, the HPD samples exhibit the highest sensory intensity, suggesting that under this drying condition, star anise retains a rich and intense aroma, primarily due to its abundance of trans-anethole [36]. Overall, the differences in the shapes and areas of the radar charts reflect variations in the composition of substances in star anise dried using different technologies.

As shown in Figure 3b, there was little difference in the aroma compounds among the samples. A total of 26 compounds were successfully identified in the samples through GC–MS analysis, as presented in Table S2. The main aroma compounds included alcohols (Eucalyptol, Isopulegol, Terpinen-4-ol, 2-p-Menthen-1-ol, Neryl alcohol, α-Terpineol, Geraniol, trans-Nerolidol, Spathulenol, Cadinol, Eudesmol, trans-Farnesol), olefins (beta-Myrcene, trans-a-Bergamotene), aldehydes (Anisic aldehyde, Cinnamaldehyde), and ether (trans-Anethole), which were consistent with the results of star anise powder reported by (Shi et al., 2021). GC-MS analysis using boiled star anise water is more reflective of the actual distribution of flavor when star anise was cooked as a seasoning. This technique shows a greater concentration of trans-anethole, the main substance in star anise, due to the enhanced intermolecular movement during the boiling process. Volatile compounds were identified at Level 1 (confirmed by reference standards and RI matching) and Level 2 (RI and mass spectrum matching to NIST14 with ≥80% similarity).

As shown in Figure 3c, two main aroma compounds in star anise water were trans-anethole and Anisic aldehyde. The trans-anethole was the key aroma compounds detected in star anise, which accounts for as much as more than 80% of the volatile oil [37]. Trans-anethole is an important raw material for the synthesis of anisaldehyde and anisole, which is widely used in food, toothpaste, soap, and cosmetic industries [38]. Among the four techniques, the highest concentration of trans-anethole brain was found under HPD, followed by HAD, and the lowest concentration was found under techniques using FIRD (*p* < 0.05). Although the visual appearance of star anise under MVD treatment was more yellow-green and not dominant, it had a faster drying speed and higher concentration of trans-anethole.

Anisic aldehyde is the second most abundant substance in star anise water, and it also has various biological activities such as antibacterial, antioxidant, sedative, and anti-inflammatory. Figure 3c shows that the content of anisic aldehyde in star anise dried using HAD and HPD is significantly higher compared to that dried with FIRD and MVD (*p* < 0.05). In addition, within the four treatment groups involving star anise water, we identified three derivatives of 2-naphthalenemethanol, each possessing similar chemical structures but differing in the positions of their substituents and stereogenic centers. These compounds are present in the volatile components of plants and exhibit certain biological activities [39]. They may be applied in multiple fields in the future, such as drug synthesis and chemical synthesis.

#### 3.4.2. Taste Release

**Figure 4:**
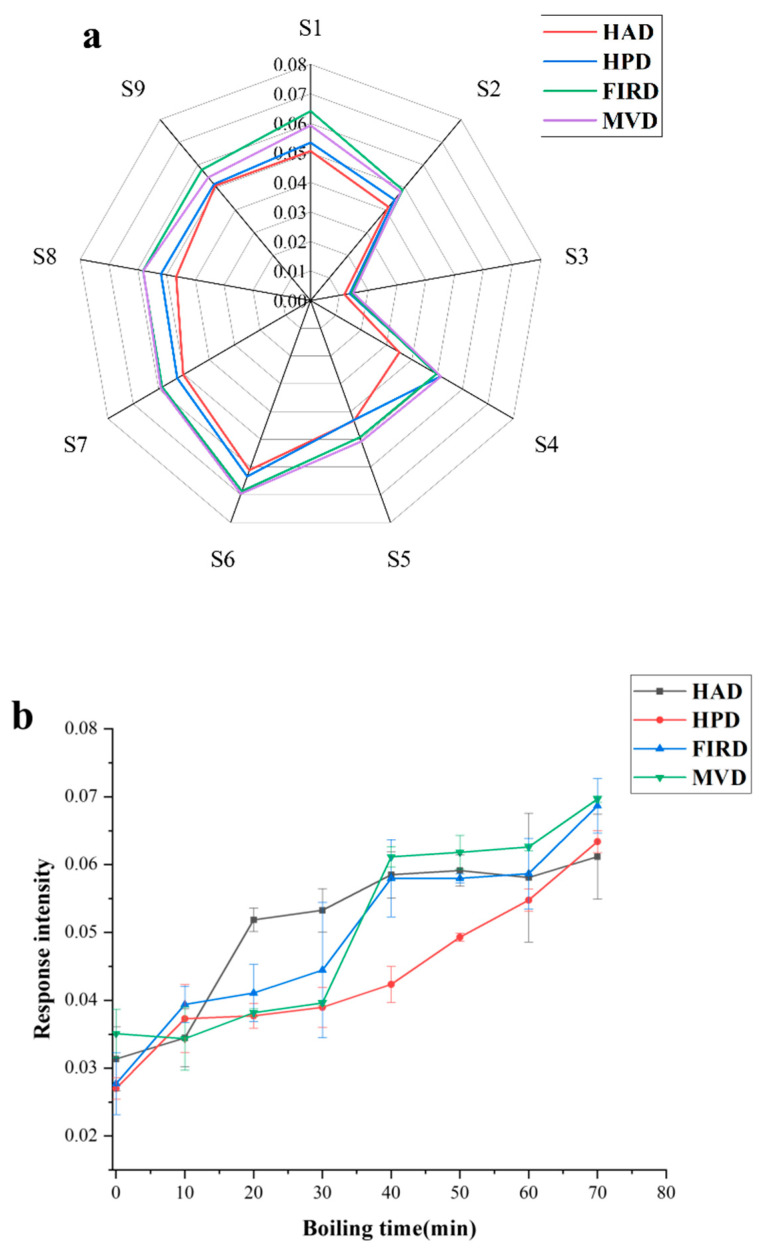
(**a**) Electronic tongue taste sensing value of star anise. (**b**) Star anise water taste release curve.

E-tongue is a biomimetic sensor technology designed to replicate the gustatory perception capabilities of the human tongue. It typically consists of multiple chemical sensors, each capable of responding to different chemical substances or characteristics [40]. Figure 4a shows the overall taste-related compounds release during the boiling of star anise treated with the four drying technologies was relatively similar. As depicted in Figure 4a, the radar response values revealed that the overall taste release during the boiling process of star anise, subjected to drying technologies, exhibited consistency across the samples. Across all drying technologies, there was a general increase in taste release from the onset of boiling 0 to 70 min [41]. Notably, MVD exhibited the highest final response value, indicating its superior ability to release taste, as shown in Figure 4b. The HPD demonstrated a smoother increase in taste release over time, while the MVD and FIRD technologies showed a surge in taste release around the 30-min mark, reaching peak values shortly thereafter. However, it is noteworthy that HAD sample showed a significant peak in taste release at the 10-min mark during boiling, after which the release intensity tended to stabilize. These results indicate that different drying methods can significantly affect the taste release pattern of star anise during boiling, possibly due to varying degrees of impact on the microstructure of star anise caused by the different drying methods, thus providing a potential technical option for controlling the flavor release of star anise.

The investigation into the flavor release of star anise during boiling, under various drying methods, offers valuable insights for its culinary applications. Samples dried using the HAD method show the most robust initial flavor release, making them ideal for quick stir-frying. Additionally, the flavor release of HAD samples remains consistently stable throughout the cooking process, rendering them perfect for hot pot dishes. In contrast, samples dried by FIRD and MVD methods exhibit a more pronounced flavor release during the intermediate stages, indicating their suitability for stewing, where a gradual infusion of flavor is preferred.

### 3.5. Water States of Star Anise Dried Using Different Technologies

**Figure 5:**
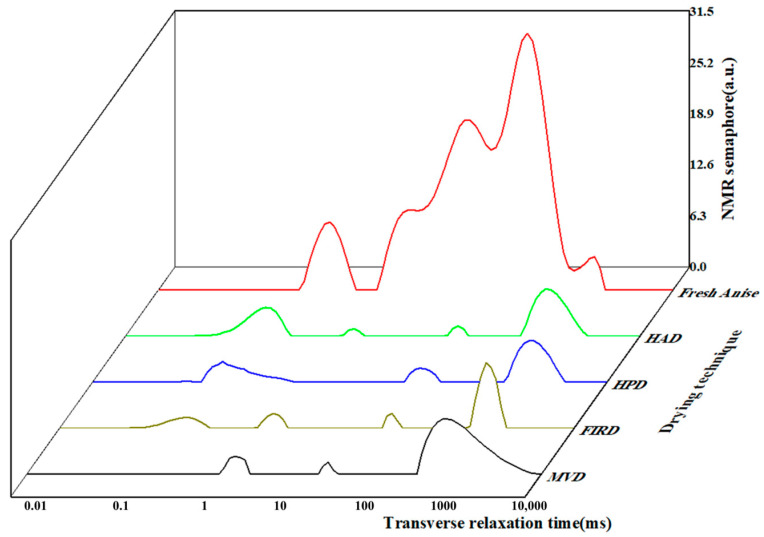
Star anise dry NMR frequency map.

The L-NMR spin-spin relaxation time curves exhibited three distinct peaks identified as *T*_21_, *T*_22_, and *T*_23_, respectively. These peaks correspond to different states of water present in the sample: *T*_21_ (0–10 ms) represents bound water tightly bound to macromolecular substances, *T*_22_ (10–100 ms) corresponds to immobilized water confined within a matrix structure, and *T*_23_ (>100 ms) indicates free water characterized by high mobility [42]. Figure 5 shows that for fresh star anise, the area of the corresponding peak (*T*_23_) in the inversion spectrum was maximal, indicating a higher proportion of free water and a relatively lower proportion of bound and less mobile water. As drying progresses, the peak amplitude gradually decreased. This phenomenon is attributed to the rapid decrease in free water content during the drying process, with a significant portion initially transforming into less mobile water and subsequently into bound water. In comparison to fresh star anise, the peaks observed in the HAD, HPD, and FIRD treatments demonstrated shifts and decreased intensities. Although all drying methods exhibited similar trends, they differed in peak positions and intensities, indicating that each technique impacts the chemical composition of star anise in distinct ways [43]. The HAD and HPD, which rely on convective heat, effectively reduce free water while retaining some immobilized water due to their milder drying conditions [44]. In contrast, FIRD, which employs far-infrared radiation, efficiently decreases free water while preserving some internal immobilized moisture due to its moderate penetration capabilities. The MVD, characterized by rapid microwave heating under vacuum, achieves the greatest reduction in water content, effectively removing both free and immobilized water due to its intense energy penetration. Notably, FIRD and MVD can directly target immobilized water, transferring it from the interior of the star anise to the exterior [45]. This may result in different alterations to the microstructure of star anise, ultimately affecting the quality parameters of the dried product.

### 3.6. Possible Mechanism on Flavor Release of Dried Star Anise During Boiling

**Figure 6:**
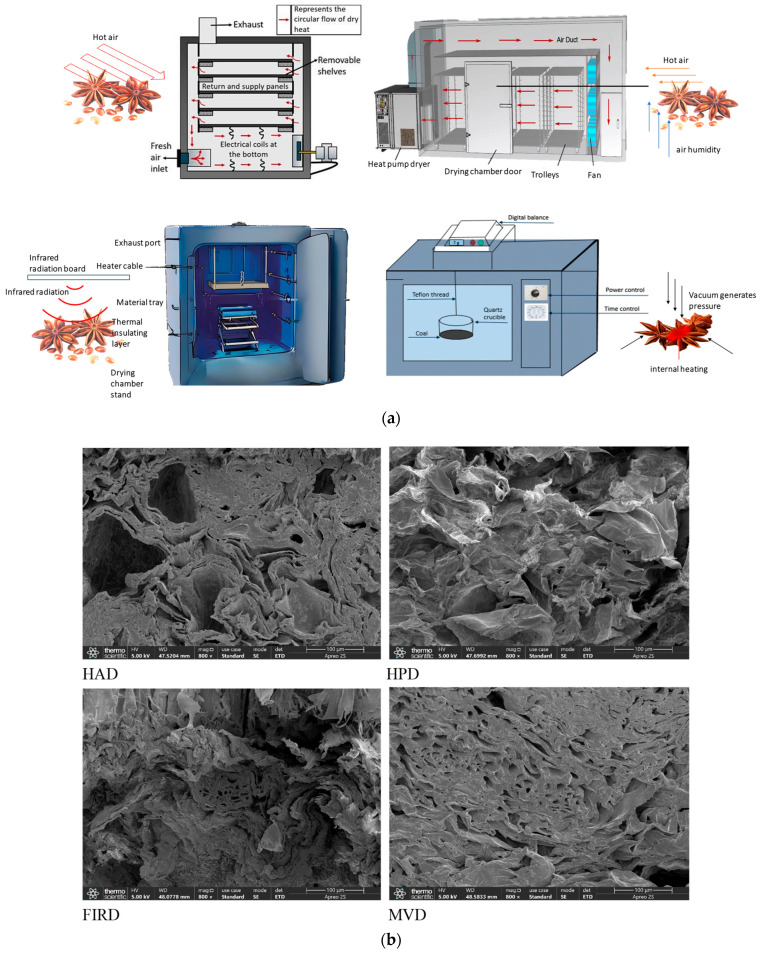
(**a**) Schematic diagram of drying mechanisms using different drying techniques. (**b**) Scanning electron microscopy of star anise.

Based on the principles of various drying techniques and the SEM analysis of star anise cross-sections at 800× magnification, a potential mechanism for flavor release during the boiling process was proposed, as illustrated in Figure 6.

In the HAD, moisture evaporation was facilitated by the circulation of hot air, making it a traditional and straightforward drying technique. However, as shown in Figure 6b, this strong convective drying resulted in the shrinkage of the microstructure of the dried octagonal fruit. During the shrinkage process, local cracking occurred, creating large voids that led to a rapid initial release of flavor, followed by a gradual plateau in subsequent stages [46]. In contrast, while both HAD and HPD were classified as conventional thermal processing methods, HPD was distinguished by its ability to not only utilize hot air for convective drying but also regulate humidity levels and exchange heat with the external environment. This multifaceted approach was found to effectively preserve thermally sensitive volatile compounds and minimize oxidation, resulting in a richer and more authentic aroma of star anise [47]. Consequently, flavor release during the HPD process was observed to be more stable.

It was also noted that the microstructures of star anise dried using the FIRD and MVD methods were quite similar, as both techniques directly affected water molecules. During the drying process, water was found to migrate from the interior of the star anise to the exterior [48]. This migration generated an internal-to-external driving force, causing some degree of expansion in the star anise structure. Since microwave drying operated at a faster rate and the vacuum environment exerted greater pressure, leading to deeper penetration, the structure of the MVD sample was observed to be fluffier compared to that of the FIRD sample [49]. This fluffier structure resulted in more comparable flavor release trends between the two drying methods. Therefore, during the boiling and flavor release process, it was concluded that the flavor compounds in samples from both drying techniques required a certain amount of time to fully migrate and be released.

While this study provides valuable insights, several considerations should be noted. The controlled laboratory conditions used may differ from commercial-scale drying operations in terms of process parameters and product consistency. Additionally, our flavor-release analysis focused specifically on boiling applications, leaving other common culinary techniques like stir-frying or roasting unexplored. Future investigations could examine scale-up parameters for industrial adaptation and systematically evaluate flavor profiles across diverse cooking methods to broaden the practical applications of these findings.

## 4. Conclusions

This study underscores the critical role of drying technology selection in optimizing both the quality and functional attributes of star anise, particularly in culinary applications. By evaluating structural, visual, and flavor-related outcomes, the research provides a framework for aligning industrial drying practices with specific consumer and industrial demands. The findings emphasize that technological choices in drying extend beyond mere efficiency, directly influencing sensory appeal and flavor dynamics during cooking—a consideration vital for enhancing product value and consumer satisfaction.

The implications of this work are particularly relevant for the food processing industry, where balancing energy efficiency with quality retention remains a persistent challenge. Future investigations should prioritize the development of adaptive drying protocols, integrating advanced technologies to mitigate trade-offs between speed and quality degradation. Additionally, exploring the interaction between drying-induced structural modifications and diverse cooking methods could unlock tailored applications, further bridging scientific research with culinary innovation. Ultimately, this study advances the understanding of how drying methodologies shape food functionality, offering a foundation for strategic improvements in spice processing and broader food-preservation practices.

## Figures and Tables

**Table 1:** Physicochemical indicators of star anise under four different drying methods (color, fracture rate, rehydration rate).

Drying Method	HAD	HPD	FIRD	MVD
fit (graph)	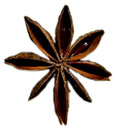	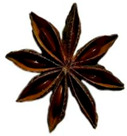	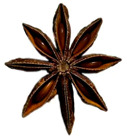	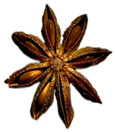
L*	25.18 ± 0.71 ^b^	24.85 ± 0.17 ^b^	25.96 ± 0.53 ^b^	30.91 ± 0.72 ^a^
a*	10.87 ± 0.81 ^b^	10.95 ± 0.48 ^b^	10.49 ± 0.88 ^b^	9.34 ± 0.51 ^a^
b*	18.06 ± 0.34 ^b^	18.32 ± 0.17 ^b^	18.44 ± 0.61 ^b^	25.03 ± 0.86 ^a^
∆*E*	68.21 ± 0.41 ^a^	68.64 ± 0.39 ^a^	67.59 ± 0.73 ^a^	64.82 ± 0.67 ^b^
Fracture rate	11.97 ± 1.07 ^a^	10.79 ± 0.56 ^ab^	9.84 ± 0.37 ^b^	10.33 ± 1.12 ^ab^

The different letters represent the significantly difference between the values after Tukey’s test (*p* < 0.05) among the values (means ± SD values).

## Data Availability

The original contributions presented in the study are included in the article/Supplementary Materials. Further inquiries can be directed to the corresponding author.

## Supplementary Materials

The following supporting information can be downloaded at: https://www.mdpi.com/article/10.3390/foods14101802/s1, Figure S1. Schematic diagram of electronic tongue detection for violates release. 1. Electronic tongue display screen; 2. Control buttons; 3. Electronic tongue power supply; 4. Detector; 5. Detection probe; 6. Electric heater; Table S1. Electronic nose sensors and main application. Table S2. Different drying methods for treating volatile compounds in star anise water. a Reliability of the identification proposal was determined: mass spectra and retention indices agreed with database or literature. b Calculated retention index (RI) on HP-INNOWAX column. c Identification methods: RI = retention index.- Not found.

## References

[1] Patra J.K., Das G., Bose S., Banerjee S., Vishnuprasad C.N., del Pilar Rodriguez-Torres M., Shin H.S. (2020). Star anise (*Illicium verum*): Chemical compounds, antiviral properties, and clinical relevance. Phytother. Res..

[2] Zou Q., Huang Y., Zhang W., Lu C., Yuan J. (2023). A Comprehensive Review of the Pharmacology, Chemistry, Traditional Uses and Quality Control of Star Anise (*Illicium verumHook. F.*): An Aromatic Medicinal Plant. Molecules.

[3] Tu D., Wu F., Lei Y., Xu J., Zhuang W., Zhao Y., Tian Y. (2024). Analysis of differences in flavor attributes of soups: A case study on shiitake mushrooms dried from different drying techniques. J. Food Compos. Anal..

[4] Feng Y.B., Suo K., Chen L.Q. (2023). Improving the hot air drying of garlic slices by perforation synergistic alcohol pretreatment. Dry. Technol..

[5] Song J., Chen Q., Bi J., Meng X., Wu X., Qiao Y., Lyu Y. (2020). GC/MS coupled with MOS e-nose and flash GC e-nose for volatile characterization of Chinese jujubes as affected by different drying methods. Food Chem..

[6] Shi Y., Chen G., Chen K., Chen X., Hong Q., Kan J. (2021). Assessment of fresh star anise *(Illicium verum Hook. f.*) drying methods for influencing drying characteristics, color, flavor, volatile oil and shikimic acid. Food Chem..

[7] Hu S., Feng X., Huang W., Ibrahim S.A., Liu Y. (2020). Effects of drying methods on non-volatile taste components of mushrooms. LWT.

[8] Hou H., Chen Q., Bi J., Wu X., Jin X., Li X., Qiao Y., Lyu Y. (2020). Understanding appearance quality improvement of jujube slices during heat pump drying via water state and glass transition. J. Food Eng..

[9] Shen C., Chen W., Aziz T., Khojah E., Al-Asmari F., Alamri A.S., Alhomrani M., Cui H., Lin L. (2024). Drying kinetics and moisture migration mechanism of yam slices by cold plasma pretreatment combined with far-infrared drying. Innov. Food Sci. Emerg. Technol..

[10] Zhao Y., Gao R., Zhuang W., Xiao J., Zheng B., Tian Y. (2020). Combined single-stage tempering and microwave vacuum drying of the edible mushroom Agrocybe chaxingu: Effects on drying characteristics and physical-chemical qualities. LWT.

[11] Lu Y.Y., Kong X.F., Zhang J.H., Guo C., Qu Z., Jin L., Wang H.Q. (2021). Composition Changes in Fruit Dried by Different Methods. Front. Nutr..

[12] Deng L.-Z., Yang X.-H., Mujumdar A., Zhao J.-H., Wang D., Zhang Q., Wang J., Gao Z.-J., Xiao H.-W. (2018). Red pepper (*Capsicum annuum* L.) drying: Effects of different drying methods on drying kinetics, physicochemical properties, antioxidant capacity, and microstructure. Dry. Technol..

[13] Bai R., Sun J., Qiao X., Zheng Z., Li M., Zhang B. (2023). Hot air convective drying of ginger slices: Drying behaviour, quality characteristics, optimisation of parameters, and volatile fingerprints analysis. Foods.

[14] Hu L., Bi J., Jin X., Qiu Y., van der Sman R.G.M. (2021). Study on the rehydration quality improvement of shiitake mushroom by combined drying methods. Foods.

[15] Chai J., Xu J., He M., Shi J., Chu J., Cui Q., Shi Q. (2024). Green recovery of nuciferine from lotus leaf via star anise oil based two-phase solvent extraction integrated with back-extraction. Sustain. Chem. Pharm..

[16] Xie Y., Guo C., Devahastin S., Jiang L., Du M., Yi J. (2024). Non-destructive determination of volatile compounds and prediction of amino acid nitrogen during sufu fermentation via electronic nose in combination with machine learning approaches. LWT.

[17] Ye Z., Shang Z., Li M., Zhang X., Ren H., Hu X., Yi J. (2022). Effect of ripening and variety on the physiochemical quality and flavor of fermented Chinese chili pepper (Paojiao). Food Chem..

[18] Yin X.Q., Zhang M., Wang S.S., Wang Z.R., Wen H.Y., Sun Z.W., Zhang Y.H. (2024). Characterization and discrimination of the taste and aroma of Tibetan Qingke baijiu using electronic tongue, electronic nose and gas chromatography-mass spectrometry. Food Chem. X.

[19] Macedo L.L., Vimercati W.C., da Silva Araújo C., Saraiva S.H., Teixeira L. (2020). Effect of drying air temperature on drying kinetics and physicochemical characteristics of dried banana. J. Food Process Eng..

[20] Nowacka M., Wiktor A., Anuszewska A., Dadan M., Rybak K., Witrowa-Rajchert D. (2019). The application of unconventional technologies as pulsed electric field, ultrasound and microwave-vacuum drying in the production of dried cranberry snacks. Ultrason.-Sonochemistry.

[21] Qu F., Zhu X., Ai Z., Ai Y., Qiu F., Ni D. (2019). Effect of different drying methods on the sensory quality and chemical components of black tea. LWT.

[22] Zhou M., Liu C., Li D. (2016). Effect of Different Drying Methods on Quality of Lotus Seeds. Food Sci..

[23] Sun Q., Yu X., Zhang L., AYagoub A.E., Tang Y., Wahia H., Zhou C. (2022). Effects of vacuum ultrasonic infiltration and combined drying on rehydration quality of ginger (*Zingiber officinale* Roscoe). Ind. Crops Prod..

[24] Ozcan-Sinir G., Ozkan-Karabacak A., Tamer C.E., Copur O.U. (2019). The effect of hot air, vacuum and microwave drying on drying characteristics, rehydration capacity, color, total phenolic content and antioxidant capacity of Kumquat (*Citrus japonica*). Food Sci. Technol..

[25] Polat A., Izli N. (2022). Determination of drying kinetics and quality parameters for drying apricot cubes with electrohydrodynamic, hot air and combined electrohydrodynamic-hot air drying methods. Dry. Technol..

[26] Xu Y., Qi J., Yu M., Zhang R., Lin H., Yan H., Li C., Jia J., Hu Y. (2023). Insight into the mechanism of water-insoluble dietary fiber from star anise (Illicium verum Hook. f.) on water-holding capacity of myofibrillar protein gels. Food Chem..

[27] Yu J., Huang D., Ling X., Xun C., Huang W., Zheng J., Zhang L. (2024). Drying kinetics of camellia oleifera seeds under hot air drying with ultrasonic pretreatment. Ind. Crops Prod..

[28] Seremet L., Botez E., Nistor O.-V., Andronoiu D.G., Mocanu G.-D. (2016). Effect of different drying methods on moisture ratio and rehydration of pumpkin slices. Food Chem..

[29] Liu J., Liu Y., Li X., Zhu J., Wang X., Ma L. (2023). Drying characteristics, quality changes, parameters optimization and flavor analysis for microwave vacuum drying of garlic (*Allium sativum* L.) slices. LWT.

[30] Shang J., Zhang Q., Wang T., Xu Y., Zang Z., Wan F., Yue Y., Huang X. (2023). Effect of Ultrasonic Pretreatment on the Far-Infrared Drying Process and Quality Characteristics of Licorice. Foods.

[31] Yu C., Zhang J., Wang T. (2021). Star anise essential oil: Chemical compounds, antifungal and antioxidant activities: A review. J. Essent. Oil Res..

[32] Xin Y.W., Yun L.C., Sheng B.L., Zheng R., Qi P. (2021). Effect of infrared radiation-hot air (IR-HA) drying on kinetics and quality changes of star anise. Dry. Technol..

[33] Cai M., Guo X., Liang H., Sun P. (2013). Microwave-assisted extraction and antioxidant activity of star anise oil. Int. J. Food Sci. Technol..

[34] Bozkir H., Tekgül Y., Erten E.S. (2021). Effects of tray drying, vacuum infrared drying, and vacuum microwave drying techniques on quality characteristics and aroma profile of orange peels. J. Food Process Eng..

[35] Pei F., Yang W., Ma N., Fang Y., Zhao L., An X., Xin Z., Hu Q. (2016). Effect of the two drying approaches on the volatile profiles of button mushroom (*Agaricus bisporus*) by headspace GC–MS and electronic nose. LWT.

[36] Chai X., Huang X., Zhang T., Wu K., Duan X., Yu H., Liu X. (2023). Comparative study of e-nose, gc-ms, and gc-ims to distinguish star anise essential oil extracted using different extraction methods. Separations.

[37] Peng Q., Luo X., Su J., Bi Y., Kong F., Wang Z., Tan S., Zhang J. (2024). Microencapsulation of star anise essential oil: Preparation, characterization, in vitro digestion, and biological activity. Colloids Surf. A Physicochem. Eng. Asp..

[38] Singh S., Verma R. (2024). Comprehensive review on pharmacological potential of Illicium verum, Chinese herb. Pharmacol. Res.-Mod. Chin. Med..

[39] Li K., Zou Y., Wang Y., Zhou M., Li J., Tan R., Zhang S., Li W., Zheng J. (2022). 2-Naphthalenemethanol participates in metabolic activation of 2-methylnaphthalene. Xenobiotica.

[40] Dong W., Hu R., Long Y., Li H., Zhang Y., Zhu K., Chu Z. (2019). Comparative evaluation of the volatile profiles and taste properties of roasted coffee beans as affected by drying method and detected by electronic nose, electronic tongue, and HS-SPME-GC-MS. Food Chem..

[41] Fan X., Zhong M., Feng L., Huo Y., Pan L. (2024). Evaluation of flavor characteristics in tartary buckwheat (*Fagopyrum tataricum*) by E-nose, GC-IMS, and HS-SPME-GC-MS: Influence of different roasting temperatures. LWT.

[42] Luo J., Li M., Zhang Y., Zheng M., Ling C.M. (2021). The low-field NMR studies the change in cellular water in tilapia fillet tissue during different drying conditions. Food Sci. Nutr..

[43] Zhu Y., Chen X., Pan N., Liu S., Su Y., Xiao M., Shi W., Liu Z. (2022). The effects of five different drying methods on the quality of semi-dried Takifugu obscurus fillets. LWT.

[44] Li M., Chen Y., Geng Y., Liu F., Guo L., Wang X. (2021). Convenient use of low field nuclear magnetic resonance to determine the drying kinetics and predict the quality properties of mulberries dried in hot-blast air. LWT.

[45] Liu Z.-L., Xie L., Zielinska M., Pan Z., Deng L.-Z., Zhang J.-S., Gao L., Wang S.-Y., Zheng Z.-A., Xiao H.-W. (2022). Improvement of drying efficiency and quality attributes of blueberries using innovative far-infrared radiation heating assisted pulsed vacuum drying (FIR-PVD). Innov. Food Sci. Emerg. Technol..

[46] Chu Q., Ren G., Duan X., Li L., Zhu K., Zhao M. (2022). Comparison of superheated steam and hot-air drying in the food industries. Food Ferment. Ind..

[47] Goh L.J., Othman M.Y., Mat S., Ruslan H., Sopian K. (2011). Review of heat pump systems for drying application. Renew. Sustain. Energy Rev..

[48] Pawar B.S., Pratape V. (2017). Fundamentals of Infrared Heating and Its Application in Drying of Food Materials: A Review. J. Food Process Eng..

[49] Ekezie C.F., Sun D., Han Z., Cheng J. (2017). Microwave-assisted food processing technologies for enhancing product quality and process efficiency: A review of recent developments. Trends Food Sci. Technol..

