# Supplementary material for: Impact of Drying-Induced Structural Modifications on Flavor Release of Star Anise During Boiling
Source: Foods. 2025 May 19;14(10):1802. doi: 10.3390/foods14101802 (PMC12110813; doi:10.3390/foods14101802)
Supplement: Supplementary file 1 [file foods-14-01802-s001.zip › foods-3619757-supplementary.pdf]

Supplementary Materials

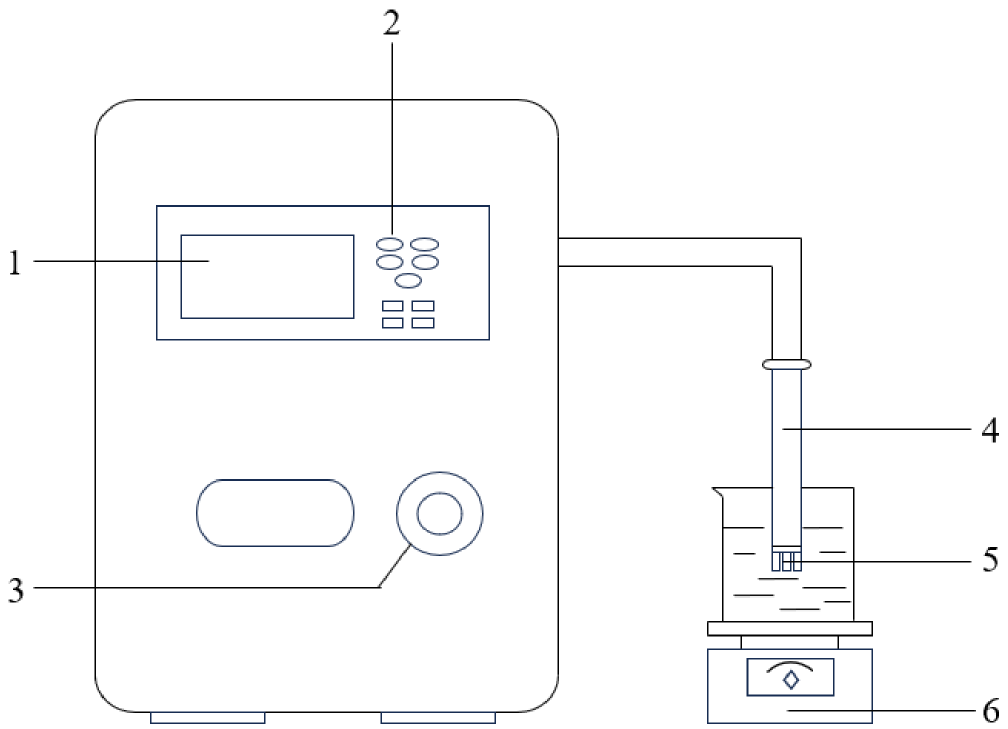

Figure S1. Schematic diagram of electronic tongue detection for violates release.

1. Electronic tongue display screen; 2. Control buttons; 3. Electronic tongue power supply; 4. Detector; 5. Detection probe; 6. Electric heater.

Table S1. Electronic nose sensors and main application**.**

Array serial No	Sensor name	Representative material specie	Performance description
1	S1	Short-chain alkanes	Propane, etc.
2	S2	Carbonaceous materials	Alcohol, smoke, isobutane, formaldehyde, etc.
3	S3	Hydrogen gas	Hydrogen, hydrogen-containing gases, etc.
4	S4	Against sulfides	Hydrogen sulfide, sulfides, etc.
5	S5	Against nitrogen-containing substances	Ammonia, amines, etc.
6	S6	Against aldehydes and ketones	Toluene, acetone, ethanol, hydrogen, etc.
7	S7	Short-chain alkane combustible gases	Methane, natural gas, biogas, etc.
8	S8	Liquefied gas	Liquefied gas
9	S9	Against alkanes, alcohols, ketones, etc.	Toluene, formaldehyde, benzene, alcohol, acetone, etc.
10	S10	Against hydrogen-containing substances, hydrogen gas, etc.	Hydrogen, etc.
11	S11	Against alkanes, carbon monoxide, etc.	Liquefied gas, alkanes, etc.
12	S12	Against certain organic solvents	Liquefied gas, methane type

13	S13	Short-chain alkane types	Methane, etc.
14	S14	Against short-chain alkanes	Methane, etc.

Table S2. Different drying methods for treating volatile compounds in star anise water.

Compounds	HAD	HPD	FIRD	MVD	RI
Bicyclo [3.1.0]hexane, 4-methylene-1-(1-methylethyl)-	-	-	0.04±0.01	-	1167
Cyclohexene, 1-methyl-5-(1-methylethenyl)-, (R)-	-	0.05±0.03	0.03±0.03	-	1200
beta.-Myrcene	-	-	-	0.02±0.01	1205
Eucalyptol	0.36±0.25[a]	0.23±0.02[ab]	0.07±0.02[b]	0.12±0.02[ab]	1210
3-Octanone	0.18±0.01[a]	0.14±0.04[a]	0.15±0.04[a]	0.11±0.04[a]	1265
Linalool	8.49±0.32[b]	9.99±1.07[ab]	10.31±1.33[ab]	10.61±0.99[a]	1556
Isopulegol	0.04±0.02[a]	0.02±0.00[ab]	0.01±0.00[b]	0.02±0.00[b]	1572
trans-.alpha.-Bergamotene	-	-	-	0.01±0.00	-
Terpinen-4-ol	2.10±1.17[a]	0.73±0.53[b]	0.71±0.31[b]	1.07±0.73[b]	1600
2-p-Menthen-1-ol	0.04±0.03	0.02±0.00	-	-	1638
4-Isopropyl-2-cyclohexenone	0.04±0.03	-	-	-	1659
Neryl alcohol	-	0.14±0.05	0.12±0.01	0.14±0.04	1700
alpha.-Terpineol	0.82±0.15	0.95±0.24	-	0.95±0.28	1808
trans-Anethole	104.86±10.45[a]	111.69±5.12[a]	82.78±5.78[b]	104.54±1.32[a]	1845
Geraniol	0.52±0.28[a]	0.53±0.16[a]	0.35±0.02[b]	0.51±0.24[a]	1856
Methyleugenol	0.01±0.00[b]	0.01±0.00[ab]	0.01±0.00[ab]	0.01±0.00[a]	2023
Anisic aldehyde	30.34±1.33[a]	30.31±0.27[a]	20.97±0.10[b]	20.78±0.29[b]	2032
Cinnamaldehyde	0.22±0.19	0.31±0.18	0.09±0.07	-	2043
trans-Nerolidol	0.29±0.14[a]	0.36±0.17[a]	0.23±0.05[a]	0.25±0.03[a]	2051
Spathulenol	0.32±0.19[a]	0.37±0.09[a]	0.27±0.06[a]	0.32±0.01[a]	2121
tau.-Cadinol	0.51±0.23[a]	0.62±0.10[a]	0.50±0.09[a]	0.60±0.09[a]	-

Continue

Compounds	HAD	HPD	FIRD	MVD	RI
gamma-Eudesmol	0.51±0.22[a]	0.41±0.04[a]	0.37±0.20[a]	0.62±0.53[a]	2170
alpha.-Eudesmol	0.36±0.22[a]	0.36±0.06[a]	0.27±0.08[a]	0.31±0.04[a]	2210
beta-Eudesmol	0.27±0.17[a]	0.24±0.01[a]	0.27±0.08[a]	0.16±0.02[a]	2220
alpha.-Cadinol,	0.76±0.48[a]	0.62±0.20[a]	0.53±0.24[a]	0.58±0.04[a]	2225
2,4-Di-tert-butylphenol	0.02±0.00[b]	0.03±0.00[a]	0.01±0.00[b]	0.01±0.00[b]	2321
trans-Farnesol	0.03±0.00[b]	0.18±0.03[a]	0.03±0.01[b]	0.02±0.00[b]	2369

a Reliability of the identification proposal was determined: mass spectra and retention indices agreed with database or literature.

b Calculated retention index (RI) on HP-INNOWAX column.

c Identification methods: RI = retention index.

- Not found.
